# Non-viral approaches in CAR-NK cell engineering: connecting natural killer cell biology and gene delivery

**DOI:** 10.1186/s12951-024-02746-4

**Published:** 2024-09-10

**Authors:** Emma M. McErlean, Helen O. McCarthy

**Affiliations:** 1 School of Pharmacy, Queen’s University of Belfast, 97 Lisburn Road, Belfast, BT9 7BL UK; 2 School of Chemical Sciences, Dublin City University, Collins Avenue, Dublin 9, Ireland; 3 Biodesign Europe, Dublin City University, Dublin 9, Ireland

**Keywords:** Cancer immunotherapy, Cell therapies, Non-viral gene delivery, Nanoparticles, Drug delivery technologies, Natural killer cells, NK-specific CAR design, CAR-NK engineering

## Abstract

Natural Killer (NK) cells are exciting candidates for cancer immunotherapy with potent innate cytotoxicity and distinct advantages over T cells for Chimeric Antigen Receptor (CAR) therapy. Concerns regarding the safety, cost, and scalability of viral vectors has ignited research into non-viral alternatives for gene delivery. This review comprehensively analyses recent advancements and challenges with non-viral genetic modification of NK cells for allogeneic CAR-NK therapies. Non-viral alternatives including electroporation and multifunctional nanoparticles are interrogated with respect to CAR expression and translational responses. Crucially, the link between NK cell biology and design of drug delivery technologies are made, which is essential for development of future non-viral approaches. This review provides valuable insights into the current state of non-viral CAR-NK cell engineering, aimed at realising the full potential of NK cell-based immunotherapies.

**Graphical Abstract:**

Non-viral production of “off-the-shelf” CAR-NK cells. 1. NK cells may be purified from donor blood, differentiated from stem cells or produced from immortalised cell lines in the lab. 2. NK-specific CAR design modified from CAR-T designs to include NK transmembrane domains (NKG2D, NKp44), co-stimulatory receptors (e.g., DAP10, 2B4) and NK cell receptors (NKG2D). 3. Non-viral genetic modification of NK cells can include delivery of CAR construct via DNA or mRNA, and knock-in/out of specific genes using gene editing tools (e.g., CRISPR Cas9, transposons). This requires a gene delivery method which may include electroporation, lipid and multifunctional nanoparticles and cell penetrating peptides. The resultant CAR-NK cells are then expanded in vitro and may be delivered as an "off-the-shelf" product to treat multiple patients.
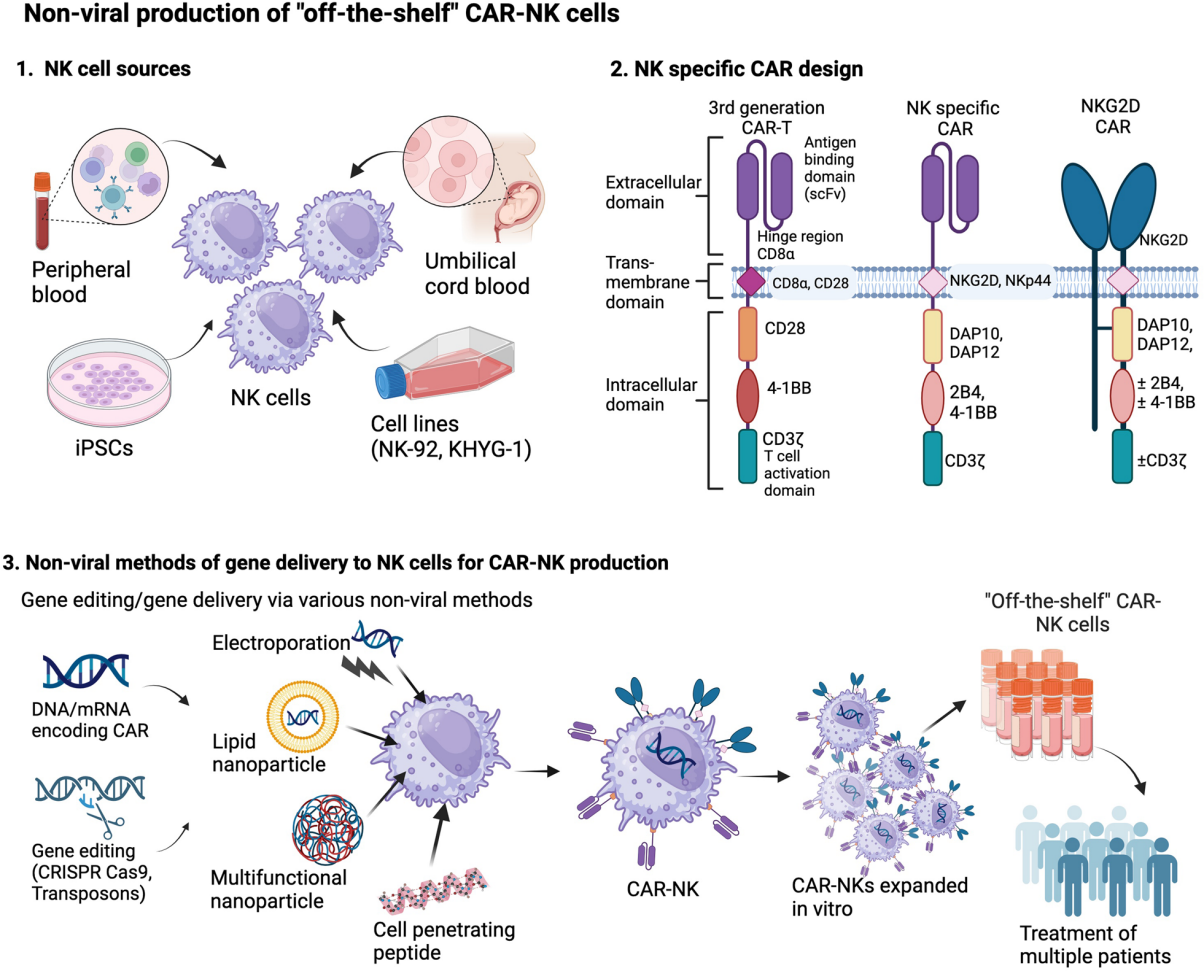

## Introduction to CAR Therapies

Chimeric antigen receptor (CAR) therapy is a type of adoptive cell therapy where T cells, dendritic cells, macrophages, or natural killer (NK) cells are genetically engineered to recognise and eliminate cancer cells [1, 2]. CAR-T therapy is the most well established and involves modifying a patient's own T cells ex vivo to express a CAR that recognises a tumour associated antigen (TAA) on the surface of cancer cells, such as CD19 which is expressed on many B-cell lymphoma and leukaemia. The CAR-T cells are then expanded and reinfused back into the patient to target and kill tumour cells expressing the specific TAA antigen [3]. The first CAR-T therapy was approved by the U.S. Food and Drug Administration (FDA) in 2017, with a further five products approved by 2022; targeting CD19^+^ malignancies including leukaemia and lymphoma, or B-cell maturation antigen (BCMA) expressing multiple myeloma (summarised in Table 1) [4–7].

**Table 1:** Details of commercially available CAR-T cell products, all of which utilise second-generation CAR design.

Product	Year of approval by FDA	Indication	Cost per treatment (USD $)	Domain structure of CAR construct					Viral vector	Source
				Antigen binding domain	Hinge region	Trans-membrane region	Co-stimulatory domain	T cell activation domain		
Tisagenlecleucel (Kymriah) from Novartis	2017	Adults with relapsed or refractory (r/r) follicular lymphoma after two or more lines of therapy	$475,000	Anti-CD19 scFV	CD8α	CD8α	4-1BB	CD3ζ	Lentivirus	[12, 13]
Axicabtagene ciloleucel (Yescarta) from Kite Pharma Inc.	2017	Adults with r/r large B-cell lymphoma (LBCL) after first-line chemoimmunotherapy.	$373,000	Anti-CD19 scFV	CD28	CD28	CD28	CD3ζ	γ retrovirus	[13, 14]
Brexucabtagene autoleucel (Tecartus) from Kite Pharma Inc.	2020	Adults with r/r mantle cell lymphoma (MCL), (r/r) B-cell precursor acute lymphoblastic leukaemia (ALL)	$373,000	Anti-CD19 scFV	CD28	CD28	CD28	CD3ζ	γ retrovirus	[15]
Idecabtagene vicleucel (Abecma) from Celgene Corporation, (Bristol-Myers Squibb)	2021	Adults with r/r multiple myeloma after four or more prior lines of therapy including an immunomodulatory agent, a proteasome inhibitor, and an anti-CD38 monoclonal antibody.	$419,000	Anti-BCMA scFv	CD8α	CD8α	4-1BB	CD3ζ	Lentivirus	[7, 16, 17]
Lisocabtagene maraleucel (Breyanzi) from Juno Therapeutics (Bristol-Myers Squibb)	2022	Adults with LBCL	$410,000	Anti-CD19 scFV	IgI4	CD28	4-1BB	CD3ζ	Lentivirus	[18]
Ciltacabtagene autoleucel (Carvykti) from Janssen Biotech Inc.	2022	Adults with r/r multiple myeloma after four or more prior lines of therapy, including a proteasome inhibitor, an immunomodulatory agent, and an anti-CD38 monoclonal antibody.	$465,000	Anti-BCMA scFv	CD8α	CD8α	4-1BB	CD3ζ	Lentivirus	[7, 19]

**Fig. 1:**
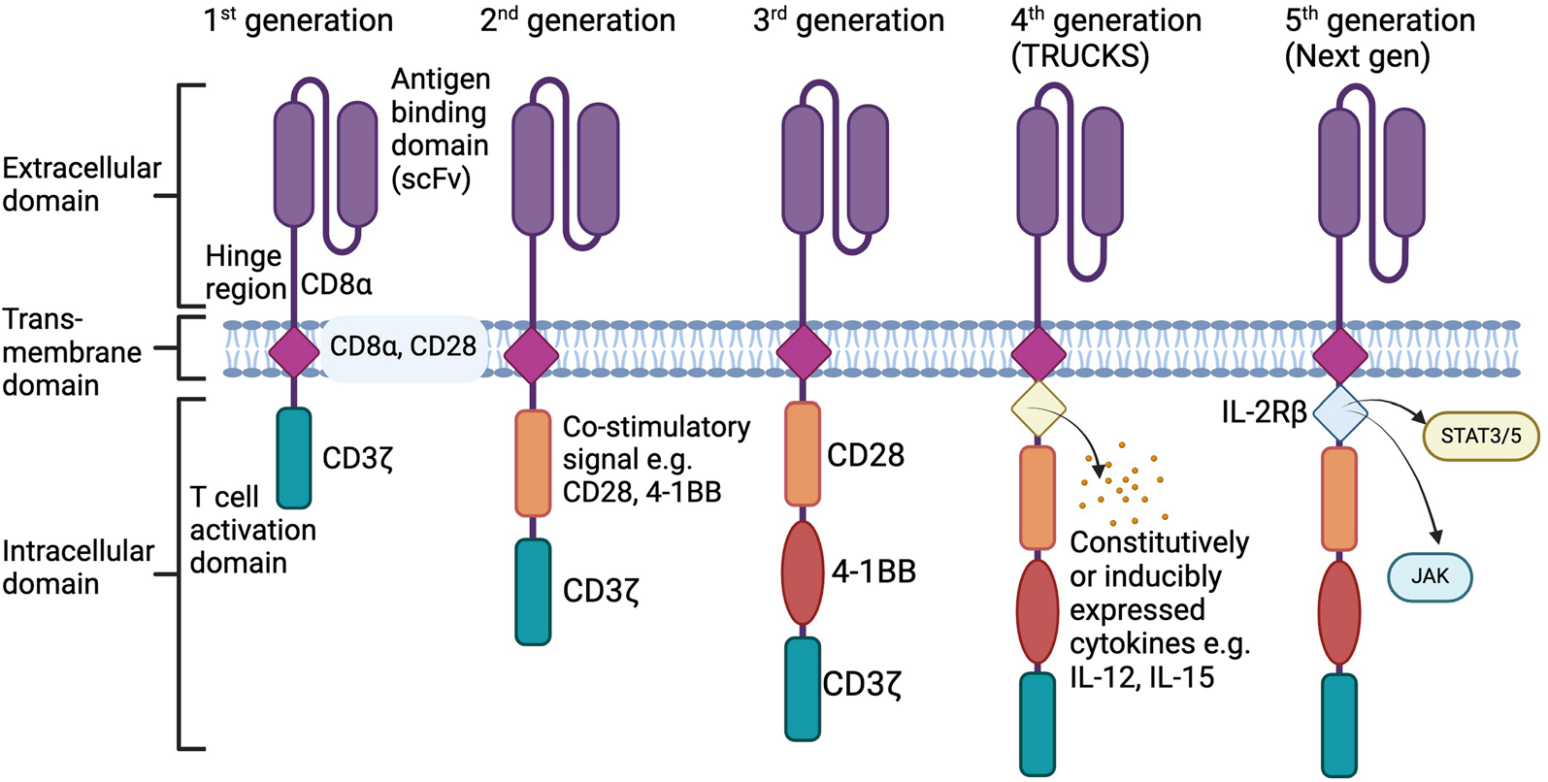
Schematic detailing the progression of CAR-T construct design elements. First generation CARs which consisted of a single-chain variable fragment (scFv) antigen recognition domain, transmembrane domain, and an intracellular T-cell activation domain derived from CD3 zeta chain (CD3ζ) progressed to second generation CAR designs, which have an additional costimulatory domain (CD28 or 4-1BB) that enhances T cell proliferation and cytotoxicity, utilised by commercial products. Further third generation CAR constructs incorporate two distinct costimulatory domains (e.g., CD28 and 4-1BB). “Armoured” fourth generation CARs (also referred to as T-cells redirected for universal cytokine-mediated killing (TRUCKs)) incorporate inducible expression components such as cytokines (e.g., IL-12) which can increase activation of CAR-T cells and tumour killing and fifth generation or “next generation” CARs contain a truncated interleukin-2 (IL-2) receptor β chain (IL-2Rβ) with a binding site for the transcription factor STAT3 to fully exploit CAR activation signals via JAK-STAT3/5 signalling; enhancing T cell activation, proliferation and persistence. Created with BioRender.com

The basic CAR structure (Fig. 1) mimics the T cell receptor activation and comprises several parts: an extracellular domain which recognises the specific antigen on the surface of cancer cells; a transmembrane domain which is critical for sending signals to the intracellular domain, activating the CAR-T cell to proliferate and survive [8]. CAR-T construct design has progressed through several generations as highlighted in Fig. 1. Commercial CAR-T cell products currently utilise second generation CAR designs, which have an additional costimulatory domain (CD28 or 4-1BB) to enhance T cell proliferation, persistence, and cytotoxicity (Table 1). Choice of costimulatory domains can significantly impact in vivo persistence; CD28-based CAR T cells persist around 30 days, compared to 4-1BB CAR T cells which may exceed 4 years in some patients [9–11].

Haematological malignancies including leukemia, lymphoma and multiple myeloma have been the main target of CAR-T therapies thus far, with impressive treatment outcomes. Between 2015 and 2017, the phase ll ELIANA clinical trial of tisagenlecleucel, with a cohort consisting of 75 paediatric patients with refractory or relapsed B cell acute lymphoblastic leukaemia (ALL), resulted in an overall remission rate of 81% at three months [20]. However, 73% experienced severe adverse events, with 47% developing severe cytokine release syndrome (CRS). Safety issues with CAR-T therapies are a major concern with patients suffering from toxicities including CRS and tumour lysis syndrome, neurotoxicity, and a risk of graft-versus-host disease (GVHD). In addition, treatment of solid tumours with CAR-T therapy has proven more difficult due to tumour heterogeneity, infiltration and immune evasion in the tumour microenvironment (TME) [21]. There are also extremely high costs associated with autologous cell therapies, with CAR-T therapies ranging from $373,000—$475,000 per treatment, and further costs associated with leukapheresis, lymphodepletion therapy, and adverse effects of CAR-T immunotherapy. For example, total costs of treatment with tisagenlecleucel per patient treated are reported to range from $478,777 for those without CRS to $531,823 for those with severe CRS, due to further treatment and hospital care requirements including intensive care treatment in some cases [13]. Consequently, the development of alternatives which are safer is warranted, to reduce the risk of complications for patients, but also help to reduce costs in terms of manufacturing and costs associated with hospital treatment required to manage severe side effects. Once such alternative is the use of Natural Killer (NK) cells for CAR therapies, which have the potential to overcome some of the problems with CAR-T therapies [22].

## Natural killer (NK) cells

**Fig. 2:**
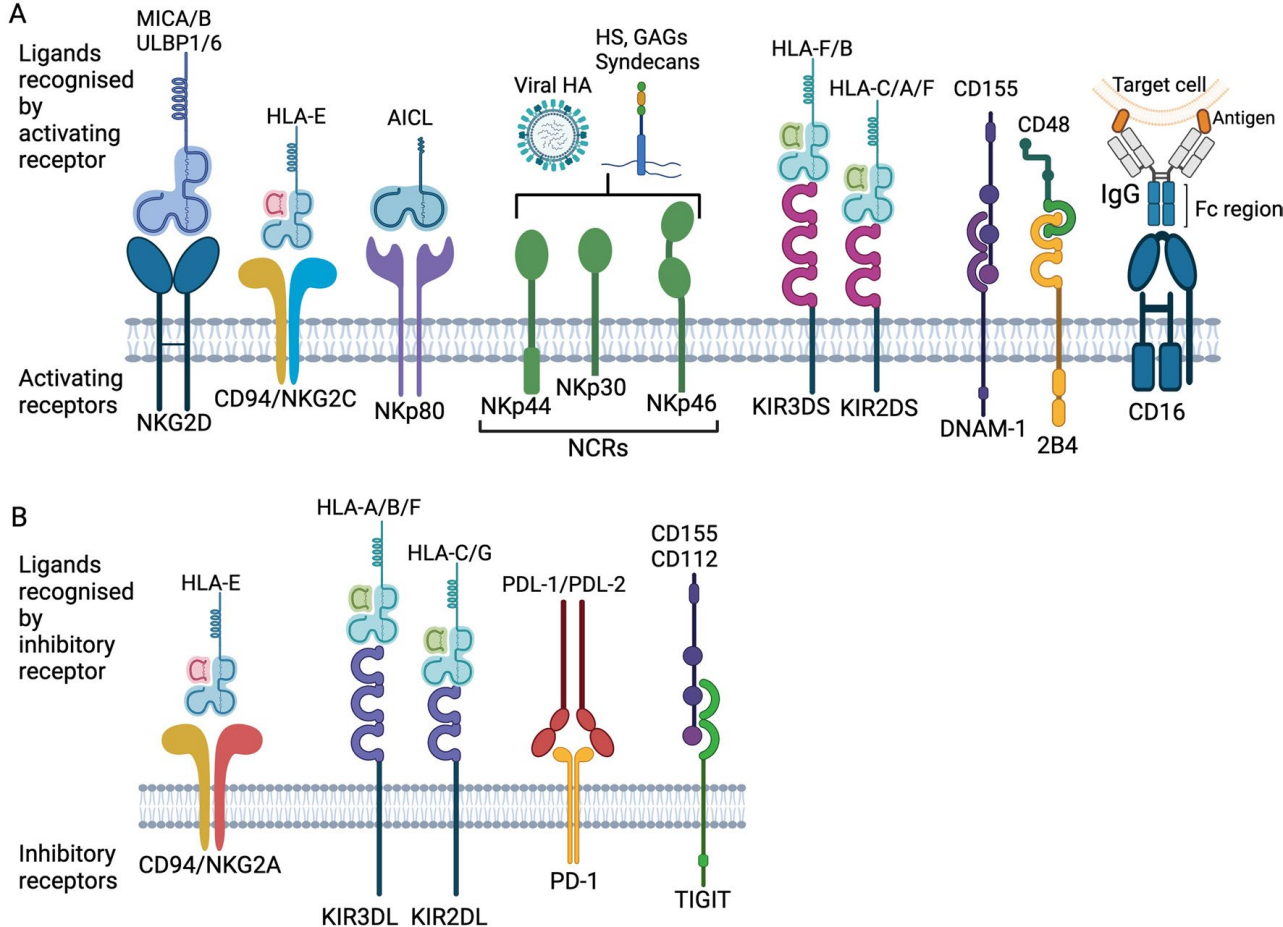
Simplified schematic of commonly expressed NK cell **A** activating and **B** inhibitory receptors and corresponding ligands. NK receptors act in synergy to provide a mechanism of self-regulation to prevent damage to healthy cells and may identify abnormal cells through downregulation of human leukocyte antigens (HLA) synonymous with major histocompatibility complex (MHC), or upregulation of stress-induced ligands, such as major histocompatibility complex class 1 chain-related protein A and B (MICA, MICB). Upon recognition of a target cell, NK cells are activated through a complex signalling pathway involving a variety of receptors, co-receptors and intracellular proteins including natural cytotoxicity receptors (NCRs), Killer Ig-like receptors (KIRs), NKG2D and DNAM-1. Created with BioRender.com

NK cells are highly cytotoxic immune effectors, defined as CD56^+^ CD3^–^ lymphocytes, capable of exerting natural cytotoxicity and antibody-dependent cellular cytotoxicity (ADCC) [23]. They play an important role in cancer surveillance with the unique ability of spontaneous cytotoxicity against cancer cells, without prior exposure or stimulation by other immune cells owing to a unique repertoire of activating receptors [24]. NK cells may identify abnormal cells through downregulation of human leukocyte antigen (HLA), synonymous with major histocompatibility complex (MHC), which are normally expressed on healthy cells, or upregulation of stress-induced ligands, such as major histocompatibility complex class 1 chain-related protein A and B (MICA, MICB). Upon recognition of a target cell, NK cells are activated through a complex signalling pathway involving a variety of receptors, co-receptors and intracellular proteins including natural cytotoxicity receptors (NCRs), Killer Ig-like receptors (KIRs), NKG2D and DNAM-1 [25–27]. Numerous NK activating and inhibitory receptors act in synergy to provide a mechanism of self-regulation to prevent damage to healthy cells which is summarised in Fig. 2 with more detail in Table 2.

**Table 2:** Summary of the main activating and inhibitory NK cell receptors which contribute to NK cytotoxic activity

Receptor family classification	Receptor name	Receptor activity	Ligand	Detail	Source
Killer Ig-like receptors (KIRs)	KIR2DL1, KIR2DL2/3, KIR2DL4, KIR2DL5, KIR3DL1, KIR3DL2, KIR3DL3	Inhibitory receptors	KIR2DL1 ligand HLA-C, group 2, KIR2DL2/3 ligand HLA-C, group 1 HLA-B, HLA-C (low affinity), KIR2DL4 ligand HLA-G, KIR3DL1 ligand HLA-Bw4, KIR3DL2 ligand HLA-A3, HLA-A11, HLA-F	Inhibitory isoforms outweigh activating ones in the affinity to HLA class I ligands KIRs are expressed at later stages in mature NK cells. Carry immunoreceptor tyrosine-based inhibitory motifs (ITIM) in the cytoplasmic domains. Provide inhibitory signalling for NK cells when interacting with their ligands (e.g., certain HLA-A, B, or C allotypes for inhibitor KIRs)	[27]
	KIR2DS1, KIR2DS2, KIR2DS3, KIR2DS4, KIR2DS5, KIR3DS1	Activating receptors	KIR2DS1 ligand HLA-C, KIR2DS2 ligand HLA-C, KIR2DS3, KIR2DS4 ligand HLA-C, HLA-A, HLA-F, KIR2DS5 ligand HLA-C, KIR3DS1 ligand HLA-F, HLA-B	All activating KIRs require DAP12 association	[27]
C-Type Lectin Family	NKG2A (CD159a) (Complex: NKG2A/CD94)	Inhibitory receptor	HLA-E	Expressed in earlier stages of NK cell differentiation (immature NK cells), prevent killing of healthy cells. Carry immunoreceptor ITIM motifs in their cytoplasmic domains. Provide inhibitory signalling for NK cells when interacting with their ligands (e.g., HLA-E for CD94/NKG2A). NKG2A forms complexes with CD94 and through the ITIM motif transduces inhibitory signals. Has a higher affinity to HLA-E ligands than the CD94/NKG2C (activating) complex.	[21]
	NKG2C (NKG2C/CD94)	Activating receptor	HLA-E	Expressed in earlier stages of NK cell differentiation (immature NK cells). Forms complexes with CD94 and associates with DAP12 for an activating signal transduction. Has a lower affinity to HLA-E ligands than the CD94/NKG2A (inhibitory receptor).	
	NKG2D (CD314) (﻿KLRK1)	Activating receptor	MICA, MICB, ULBP-1-6	Also known as KLRK1 (killer cell lectin-like receptor subfamily k, member 1). Recognises/binds to “kill me” stress signals expressed on tumour cells: Major histocompatibility complex class 1 chain-related protein A and B (MICA, MICB) and (UL16 binding protein) ULBP-1 - ULBP-6. Activating receptor responsible for provoking caspase-mediated apoptosis.	[26]
	NKp80 (KLRF1)	Activating receptor	Activation inducted C-Type Lectin (AICL), also known as CLEC2B	AICL is preferentially expressed on the surface of myeloid cells. The NKp80/AICL interaction promotes myeloid cell killing and stimulates IFN-γ cytokine release from NK cells and TNF from myeloid cells. It cooperates in the NK-mediated killing of phytohemagglutinin (PHA)-stimulated blast cells but not of various tumour cells.	[25]
Natural Cytotoxicity Receptors (NCRs)	NKp30 (NCR3) (CD337)	Activating receptor	Viral hemagglutinin (HA), Heparan sulfate (HS), glycosaminoglycans (GAGs), ﻿pp65, B7-H6, galectin-3, BAG6, ﻿(DBL)-1a domain of Plasmodium falciparum erythrocyte membrane protein-1, B7-H6 immunoligand on tumour cells	Play a major role in tumour cell recognition and killing. Recognise a diverse set of viral, oncogenic, or stress-induced ligands. Recruit immunoreceptor tyrosine-based activation motif (ITAM)-bearing adapter molecules DAP12, CD3z or FcRIg, which in turn initiate activation signalling cascades. NKp30 recognises B7-H6 immunoligand. B7-H6 also has links with programmed cell death protein (PD-1) - implications for immune checkpoint inhibitors. Expressed constitutively on mature, resting or activated NK cells and some populations of T cells. Downregulated on memory-like NK cells.	[21, 28, 29]
	NKp44 (NCR2) (CD336)	Activating receptor	HA, HS, GAGs, Platelet-derived growth factor-DD (﻿PDGF-DD), Exon 21 specific mixed lineage leukaemia protein-5 (21spe-MLL5), proliferating cell nuclear antigen (PCNA), Syndecan-4, Nidogen-1	Found only on activated NK cells. Associates with the DAP12 homodimer for cytokine release ignition.	
	NKp46 (NCR1) (CD335)	Activating receptor	HA, HS, GAGs, Complement factor P, (DBL)-1a domain of Plasmodium falciparum erythrocyte membrane protein-1, vimentin	Uniquely expressed on NK cells. Triggers cytotoxic reaction upon activation.	
Co-receptors	2B4 (CD244)	Activating receptor	CD48	The 2B4 receptor (SLAMF4, CD244) is a member of the signalling lymphocyte activation molecule (SLAM), which is a part of the immunoglobulin (Ig) superfamily. Interacts with CD48 (SLAMF2) and has an activating function that increases cytolytic function in NK cells CD48 is a glycophosphatidylinositol (GPI)-linked member of the CD2 family and is expressed on all hematopoietic cells e.g. melanoma cells Evidence suggests activation of 2B4 is dependent upon simultaneous engagement of a main activating receptor e.g. NCRs	[30]
	DNAM-1	Activating receptor	CD112, CD155	Recognition of CD112 (Nectin-2) and CD155 (Poliovirus receptor PVR) on tumour cells. Expressed on NK cells, T cells and monocytes. Signalling promotes cellular adhesion, actin and granule polarisation.	
Immune checkpoint markers	CD96	Activating	CD155	Interacts with CD155 on tumour cells	[28]
	TIGIT (T-cell immunoglobulin and ITIM domain)	Inhibitory	CD112, CD113, CD155	Interacts with CD155 and CD112 on tumour cells. NK cells with low levels of TIGIT expression show higher cytokine secretion capability, degranulation activity, and cytotoxic potential than NK cells with high levels of TIGIT expression	[31, 32]
	PD-1	Inhibitory	PDL-1, PDL-2	Expressed on various immune cells. Binds to ligands present across multiple cancer types leading to NK cell exhaustion and cancer immune escape. Most abundantly expressed by mature NK cells	[29]

**Fig. 3:**
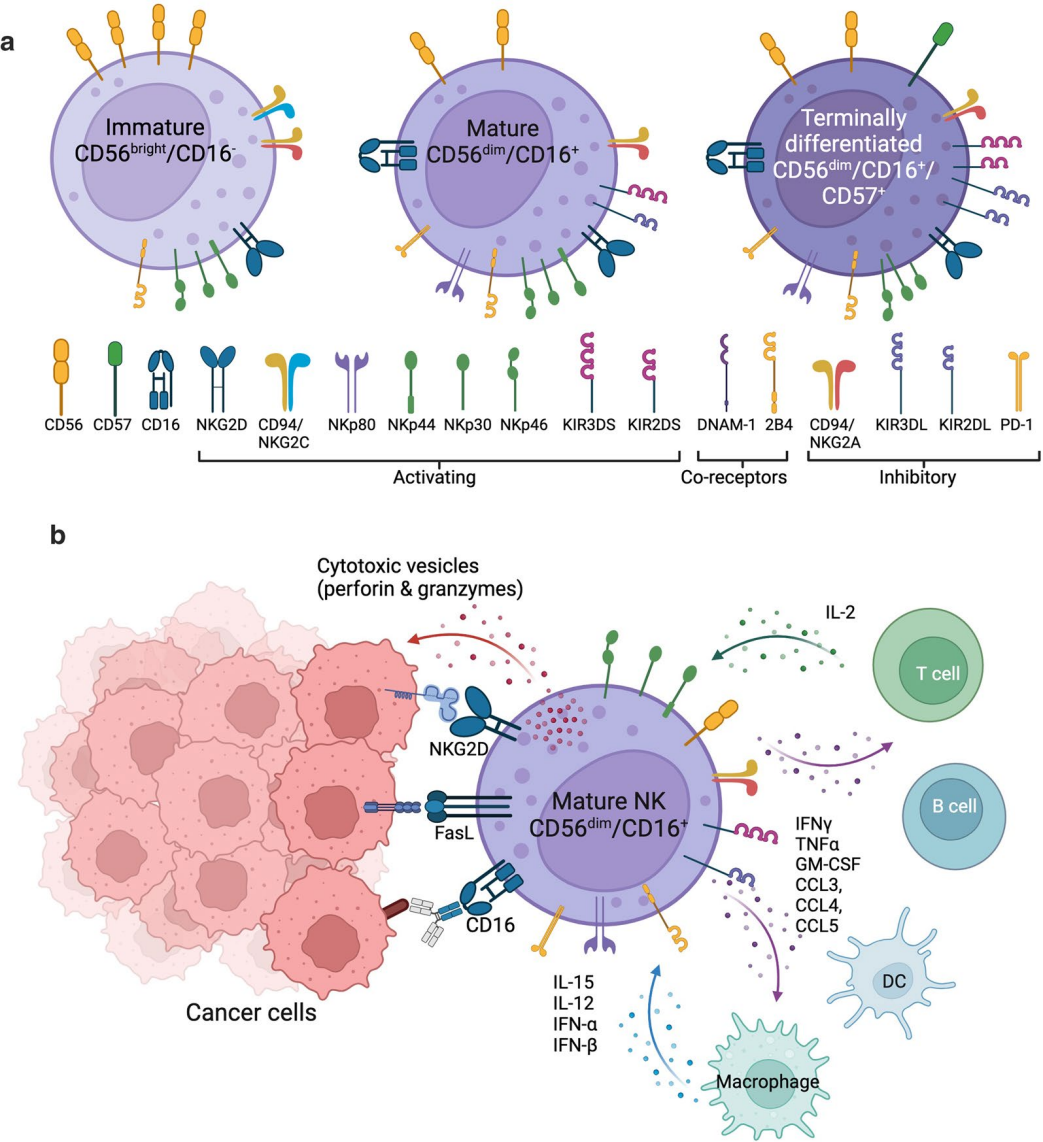
NK cell maturation and interaction with other immune cells. **A** Changing receptor status according to maturation state of NK cells from immature CD56^bright^/CD16^−^ cells to mature CD56^dim^/CD16^+^ and terminally differentiated NK cells which also may be described as “memory-like” NK cells. **B** NK cell signalling and interaction with myeloid cells and target cells following activation. NK cells mediate cytotoxicity against target cells via direct killing (activating receptors including NKG2D, NKp30, NKp44, NKp46, KIRs), apoptosis (TRAIL and FasL death receptors) and/or ADCC (CD16) and produce inflammatory cytokines and chemokines which stimulate and recruit other immune cells. Created with BioRender.com

Inhibitory receptors recognise HLA molecules, enabling NK cells to distinguish between normal and abnormal cells. When the balance tips in favour of activating receptors, cytotoxic granules are released containing perforin and granzyme B to cause direct killing through caspase-mediated apoptosis of the target cells [33]. NK cells also express death receptors, such as FasL, TRAIL and tumour necrosis factor-alpha (TNF-α) receptors, to induce apoptosis in target cells expressing the corresponding receptor [34, 35]. A variety of cytokines and chemokines are produced, such as interferon-gamma (IFN-γ) and TNF-α, causing activation of other immune cells including dendritic cells, macrophages and T cells, and promoting inflammation [31, 36, 37]. Furthermore, NK cells are capable of ADCC through recognition of target cells coated with antibodies. Mature NK cells express the CD16 receptor (also named FcγRIIIA) which recognises the Fc fragments of an immunoglobulin G (IgG) bound to an epitope on the surface of a target cell [25]. Following interaction with IgG coated cells, immunoreceptor tyrosine-based activation motif (ITAM)-containing subunits of CD16 connect the receptor to intracellular signal transduction pathways which triggers reorganisation of actin and microtubules to enable ADCC. This culminates in release of cytotoxic granules containing perforin and granzyme, engagement of FasL and TRAIL death receptors and release of cytokines and chemokines which promote further recruitment of a variety of immune cells [38]. The expression of CD16 is pivotal for NK cell ADCC function and is expressed on more mature NK cells. CD16 expression is therefore used in the nomenclature to classify the maturation state of NK cells. Human NK cells are conventionally sub-divided into two major subsets: CD56^bright^CD16^dim/−^ and more mature CD56^dim^CD16^+^. The CD56^dim^ subset are more predominant in peripheral blood, while CD56^bright^ are more abundant in tissues. CD56^bright^ NK cells are regarded as immature, expressing NKG2A but not KIRs. They are poorly cytolytic, secrete cytokines IFN-γ, GM-CSF, and proliferate in response to IL-2 or IL-15. Conversely, CD56^dim^ are regarded as mature, express NKG2A and/or KIR receptors and have strong cytolytic activity and cytokine secretion upon activation. The most mature, terminally differentiated NK cells are classified as CD56^dim^ KIR^pos^ CD57^pos^ CD16^bright^ (Fig. 3) [23, 39]. The mature CD56^dim^CD16^+^ subset is ideal for cancer therapy due to strong cytolytic activity and ADCC capacity. Consequently, adoptive immunotherapy with NK cells has emerged as a promising treatment option and research is focusing on investigating ways to harness the power of NK cells for use in cancer immunotherapy, such as CAR-NK cell therapy.

**[**FIGURE 3**].**

### Advantages of CAR-NK therapies

NK cells have several potential advantages over T-cells for CAR therapies including enhanced safety profile, potential for allogeneic therapy and wider target specificity through CAR-dependent and CAR-independent cancer cell targeting. CAR-Ts secrete a range of cytokines including IL-1, IL-2, IL6, TNF-α, MCP1, IL8, IL10, IL-15. These cytokines, and in particular the pro-inflammatory TNF-α, IL-1 and IL-6, are reported to be responsible for the severe side effects associated with CAR-T therapies including neurotoxicity and CRS [40]. In contrast, a different cytokine profile is associated with CAR-NK, which secrete IFN-γ and GM-CSF [41]. Indeed, no increase in inflammatory cytokines were detected over baseline following administration of CAR-NK cells in patients with CD19^+^ lymphoid tumours [42]. This difference in cytokine release profile is proposed as one reason for the enhanced safety profile with NK cells resulting in less toxicity and reduced cytokine storm occurrence. Although it is important to note these conclusions are based on only a small number of clinical CAR-NK studies with limited patient numbers, compared to numerous large CAR-T clinical studies, and no direct comparison has yet been carried out between CAR-T and CAR-NK therapies. Another potential safety advantage with CAR-NK cells is the reduced risk of on-target, off-tumour toxicity which has been associated with CAR-T cells [43]. All cells, malignant and non-malignant, such as CD19^+^ B cells, which express the CAR-target antigen may be targeted by CAR-T cells. The inhibitory receptors on NK cells may act as a safety switch if non-malignant cells are recognised by the CAR antigen. NK inhibitory signals would be initiated by recognition of non-malignant cells, preventing off-tumour toxicity [44].

The potential for allogeneic application of NK cells lies in the low risk of GVHD following CAR-NK cell therapy. In the case of T cells, with their highly variable αβ T-cell receptors (TCRs), donor T cells bind with HLA class I molecules widely expressed on recipient tissue cells. Following infusion of donor T cells, the TCR recognises the HLA as foreign, initiating an immune response against recipient cells, causing an alloreaction leading to GVHD [45]. In comparison, NK activity is not dependent on HLA I recognition, allowing NK cells to target malignant cells which often lose their HLA l receptor as a means of immune evasion [5]. As a result, the risk of GVHD is low with NK cell therapies [46], and studies on NK cells have produced promising results in relation to safety.

Due to the improved safety profile and lower risk of GVHD, there is potential for the development of an “off-the-shelf” CAR-NK product [47], which could be transformative for the field. Although autologous NK cell therapies are under investigation, allogeneic NK cells may be more effective than autologous NK therapies, overcoming issues with exhausted NK cells from immunosuppressed patients [48]. Allogeneic donor NK cells can be grown in large batches, facilitating more efficient quality control testing, transfection, and infusion [49]; allowing for treatment of multiple patients from the same batch which could negate the need for specialist centres. In contrast, T cells used in current CAR therapy are typically derived from the patient's own cells, which can be time-consuming and costly to harvest and engineer. Autologous CAR-T therapy can cost up to $475,000 USD per treatment, due to CAR-T therapy being labour intensive, difficult to scale and prone to failure [13]. In comparison, the cost of a cycle of an allogeneic “off-the-shelf” NK-92 treatment could be less than $20,000 USD, making it a much more cost-effective option [50, 51]. It is important to note that such costs savings are only achievable when engineered allogeneic NK-cell products are shared among multiple patients. Furthermore, efforts to produce allogeneic T cells are ongoing and could also serve to overcome the problems encountered with autologous T cell engineering.

CAR-NK therapies may have potential in treatment of solid tumours. CAR-T therapies have produced disappointing treatment outcomes in solid tumours due to barriers such as tumour heterogeneity, immunosuppressive tumour microenvironment (TME), insufficient trafficking and infiltration, and toxicities [21]. CAR-NK cells have been investigated in a wide range of solid tumours such as glioblastoma, small-cell lung, breast, prostate, ovarian, colorectal and pancreatic cancer, and it is proposed that the natural properties of NK cells may be beneficial in treating solid cancers [52]. In contrast to lymphomas with stable overexpression of B-cell antigens, solid tumours often have variable antigen surface expression levels and heterogeneity within tumours and between primary and metastatic tissue makes successful treatment difficult with a single CAR target [53]. In addition to any CAR modification, NK cells have the potential to recognise heterogenous tumour cells via multiple activating and inhibitory receptors despite the diminished or absent expression of HLA-I molecules. The presence of activating signals, such as NKG2D, allow NK cells to exert their cytotoxicity directly against transformed cells which lack the specific CAR antigen and would otherwise be left untouched by CAR-T cells and is often the case in the heterogenous solid tumour environment. This is a distinct advantage over CAR-T cells which are only active against cells expressing the CAR antigen and allows NK cells to target a wider cancer cell population. Indeed, even without the addition of CAR-antigens, unmodified NK cells have been utilised to target cancer cells and have shown early signs of their effectiveness in clinical trials [54]. Furthermore, in addition to their direct cytotoxic effects against cancer cells, NK cells are also important for recruiting conventional type 1 dendritic cells (cDC1) and subsequently CD8^+^ T cells which would promote the cancer immunity cycle activation, with the potential of overcoming the immunosuppressive TME [55].

## Current state of CAR-NK therapy development

Currently there are no approved NK cell-based products on the market, but the number of clinical trials investigating NK cell therapies has exploded since 2016, with nearly 60 trials initiated by May 2023 [48]. The field is still only recently emerging, with the large majority of trials at phase I or II and still ongoing, thus results are not fully reported yet. However, early data has been promising and suggests that CAR-NK cell therapy is safe and has the potential to be an effective treatment for cancer, although significant clinical effects have yet to be reported. A range of antigens and malignancies are targeted but a significant number of CAR-NK trials focus on targeting CD19 for haematological cancers, which has been a successful target of CAR-T therapy.

One example of CAR-NK cells targeting CD19, is the phase I/II clinical trial investigating the safety and efficacy of CD19-targeted CAR-NK cells in patients with relapsed or refractory CD19^+^ B-lymphoid malignancies including ALL, chronic lymphocytic leukaemia (CLL) and non-Hodgkin’s lymphoma (NCT03056339) [42, 56]. HLA-mismatched NK cells derived from cord blood were co-cultured with K562 feeder cells and IL-2 and transduced on Day 6 with a retroviral vector expressing anti-CD19 CAR, IL-15 to enhance in vivo expansion and persistence of CAR-NK cells and inducible Cas9 as a safety switch to trigger apoptosis of CAR-NK cells in the event of unacceptable toxic effects. Following expansion, the CAR-NK cells were harvested for fresh infusion on Day 15 and administered to 37 patients with relapsed or refractory CD19^+^ cancers including non-Hodgkin’s lymphoma and CLL. Clinical responses to therapy were based on the 2018 criteria of the International Workshop on CLL (iwCLL) and the 2014 Lugano classification for non-Hodgkin’s lymphoma [57, 58]. The 1-year overall survival was 68% with progression-free survival reported as 32%. Importantly, no significant toxicities occurred including CRS, neurotoxicity or GVHD, and there was no increase in the levels of inflammatory cytokines including IL-6. The promising clinical outcome and safety profile highlights the potential of CAR-NK therapy as an allogeneic therapy which could be developed into an “off-the-shelf” product.

The potential for CAR-NK cell therapies to treat solid tumours has driven investigation in this area and several clinical trials are ongoing in glioblastoma, small-cell lung, breast, prostate, ovarian, colorectal and pancreatic cancer [43, 52]. Many of these studies are in the very early stages and still recruiting patients, so results have not been fully reported yet. Recently, Burger et al. published the results of the first clinical trial to explore adoptive CAR-NK cell therapy in glioblastoma patients; a phase I trial investigating intracranial injection of human epidermal growth factor receptor 2 (HER2)-targeted CAR-NK cells during relapse surgery in 9 patients with recurrent HER2^+^ glioblastoma (NCT03383978) [59]. This study investigated lentivirally transduced NK-92 cells expressing a CAR comprising a HER2-specific scFv antibody, a CD8a α hinge regions and CD28 and CD3ς signalling domains. Following injection of the CAR-NK cells into the margins of the surgical cavity during surgery, five patients showed stable disease that lasted 7 – 37 weeks. Four patients had progressive disease and pseudo-progression was found at injection sites in two patients, suggestive of a treatment-induced immune response. For all patients, median progression-free survival was 7 weeks, and median overall survival was 31 weeks. Furthermore, there were no dose-limiting toxicities, and none of the patients developed CRS or immune effector cell-associated neurotoxicity syndrome. This study demonstrated the feasibility and safety of CAR-NK cells for treatment of glioblastoma. Although this was a very invasive study requiring injection of the CAR-NK cells during surgery, further studies are required to assess the ability of CAR-NK cells to target and infiltrate solid tumours following IV infusion. It is hoped that these promising results will also be replicated in other solid tumour types, in which early stage clinical trials are underway.

## CAR-NK production considerations

Despite the promising safety and therapeutic outcomes, CAR-NK therapies are still in their infancy and much work is to be done before they are approved for use in the clinic. Some considerations that need to be addressed if the potential of CAR-NK therapies is to be realised include the source of NK cells, CAR construct design and genetic modification methods for NK cells.

### Allogeneic NK cell sources

Allogeneic NK cells may be obtained from several sources including peripheral blood (PB), umbilical cord blood (CB), inducible pluripotent stem cells (iPSCs) and immortalised cell lines. Each of these sources have unique considerations for expansion and genetic modification and all are currently under clinical investigation.

#### NK cells derived from peripheral blood (PB) and cord blood (CB)

Primary NK cell may be derived from PB or CB sources. PB NK cells are the most accessible method of obtaining NK cells for CAR therapy and are widely utilised for NK cell applications. However, cell proliferation can be low with a short cell survival time [60]. Approximately, 85–95% of PB NK cells are CD56^dim^CD16^high^ which are highly cytotoxic, but less proliferative than their cytokine producing CD56^bright^ counterparts (5–15% of PB NKs) [61]. In comparison, CB-derived NK cells may be more easily obtained due to routine banking, and are more proliferative than PB NK cells, allowing for ease of expansion in vitro. However, CB NKs are phenotypically and functionally immature and may not be as cytotoxic are NKs derived from other sources [62–64].

Following isolation of NK from PB or CB, expansion is required for creation of allogeneic doses which has proven challenging due to the large number of NK cells required for therapeutic doses. Expansion protocols may be classified as feeder-cell-based or feeder-free systems. Co-culture with feeder cells (e.g. Epstein-Barr Virus transformed lymphoblastic cell lines (EBV-LCL)) which present ligands to NK cells, stimulates activation and expansion of NK cells to large numbers relatively quickly (within a few weeks). However, the use of feeder cells is associated with safety and regulatory concerns including the potential for residual viable feeder cells in the final NK cell product [64]. Consequently, feeder-free expansion methods using cytokines and antibodies to stimulate NK cells have been developed. Cytokines including IL-2 and IL-15 are potent NK cell stimulants, but a range of cytokine combinations including IL-15, IL-21, IL-12, IL-18 and IL-27 have shown promise for NK expansion [65]. While feeder-free systems avoid the safety concerns associated with feeder cells, the resultant NK yields are lower. A combination of feeder cells genetically modified to express stimulating cytokines has resulted in highly cytotoxic and proliferative NK cells. For example, K562 feeder cells genetically modified to express membrane-bound IL-15 and 41BB ligand (K562mbIL12-41BBL) results in large scale expansion of PB NK cells within 10 days of culturing in gas-permeable static cell culture flasks (G-REX) [66]. Another factor which can impact on the potency of NK cells is cryopreservation. NK cells derived from PB or CB sources may be cryopreserved before expansion or in the case of “off-the-shelf” products, storage may be required before infusion into patients. This can have detrimental effects on NK proliferation and cytotoxic functionality. Potential solutions to this include preparation of excess cells to allow for loss following cryopreservation or stimulation with cytokines such as with IL-2 immediately after thawing or pretreatment with IL-15 and IL-18 to enhance viability after cryopreservation [64, 67]. Furthermore, in the context of non-viral genetic engineering methods, PB and CB NK cells remain difficult to manipulate compared to iPSCs and cell lines.

Factors relating to the culture and expansion of NK cells may impact on transfection rates including activation state of NK cells, timing of transfection during expansion, method of expansion (feeder or feeder-free methods) and cryopreservation. Viral transduction rates are enhanced in NK cells which have been expanded (activated) ex vivo, with lower transduction observed in resting primary NK cells [68]. Standard protocols for large scale expansion of NK cells require an initial expansion before viral transduction, which is then followed by a second stage of expansion [66]. This protocol of sequential primary expansion, genetic editing and secondary expansion is also required for electroporation, and the method and timing of expansion based on feeder-cells or feeder-free systems may also impact transfection rates. Feeder cells may enhance genetic editing via induction of cell division and feeder stimulation may support NK cell recovery following ex vivo manipulation [69]. For example, Pomeroy et al. observed highest transposition of TcBuster following electroporation on Day 4 of co-culture of PB NK cells with K562-mbIL-21-41BB feeder cells. The ratios of feeder cells to NK were also adjusted depending on the stage of protocol (2:1 feeder:NK prior to electroporation, 5:1 48 h after electroporation or 1:1 for all subsequent expansions) [70]. Similarly, Gurney et al. used EBV-LCLs (10:1 feeder:NK ratio) to activate NK cells in the presence of IL-2 and IL-21. NK cells were electroporated on Day 4 and rested for 48 h in presence of IL-15 before restimulating with feeder cells [71]. Further expansion was carried out up to Day 25 with variation in the expansion of cells observed between donors. This highlights the complexity of optimisation required for genetic engineering and expansion of NK cells and a wide range of factors may impact the transduction/transfection rates, with no standard protocols established yet. Further research is required to elucidate optimal conditions and it may be necessary to tailor conditions depending on the gene delivery method.

#### iPSC derived NK cells

An alternative to the issues with primary PB and CB derived NK cells is iPSCs, which offer an unlimited source to produce homogenous NK cells and can be genetically modified and expanded on a large scale [64]. iPSC-derived CAR-NK cells can be generated from a single iPSC line and then differentiated and expanded into a universal cell product, with potential for an “off-the-shelf” therapy. In December 2021 a Fate Therapeutics press release detailed promising interim clinical data on a phase I study investigating “off-the-shelf” iPSC derived CAR-NK cells expressing CD19, termed FT596, for treatment of relapsed/refractory B-cell Lymphoma (NCT04245722) [72, 73]. FT596 iPSC CAR-NK cells were engineered to express a high affinity non-cleavable variant of CD16 for enhanced ADCC; a membrane-bound IL-15/IL-15R fusion protein to support in vivo persistence; and an NK cell optimised anti-CD19 CAR for direct tumour targeting [74]. It was reported that 10 of 11 patients treated with a second FT596 cycle continued in ongoing response, with three patients in ongoing complete response at ≥ 6 months follow-up. Furthermore, FT596 treatment regimens were well-tolerated with no dose-limiting toxicities, such as neurotoxicity or GVHD, although there were two low-grade adverse events of CRS reported in the FT596 single-dose escalation cohort which resolved without intensive care treatment. This early data highlights the potential for iPSC CAR-NK therapy, but further studies and optimisation will need be completed before such treatment is rolled out at a larger scale in the clinic.

One issue with iPSC NKs is the requirement for highly specialised culture conditions, where iPSCs are differentiated into CD34^+^ hematopoietic progenitor cells before further differentiation into NK cells. Well established protocols for producing NK cells from iPSC can take 4–5 months, although more recently a new method has been developed with reduced processing time of around 2 months [75]. Controlled differentiation of iPSCs is a labour-intensive manufacturing process requiring genetic engineering and cell culture expertise, which could be problematic for large scale manufacture of an “off-the-shelf” product which is financially viable and accessible to a broader patient population [74, 76]. In addition, major concerns exist regarding the risk of genetic mutations with iPSCs, particularly when long culture periods are required. Furthermore, there is a risk of potential tumorigenicity from undifferentiated iPSCs [77]. Therefore, there is a need to develop robust quality testing on iPSC-derived cells including screening for any genetic alterations which may have occurred during culturing.

#### NK cell lines

To date there are 10 immortalised model NK cell lines, including NK-92, KHYG-1, NKL and YT, which have been established from NK/T lymphoma patients and are used pre-clinically for the development of CAR therapies [48]. NK cell lines are highly cytotoxic toward malignant cells and have an advantage of being more easily genetically modified and expanded than NK cells from primary PB or CB sources [78–81]. Although it is important to note NK cells lines are known to be more difficult to culture than other types of cell lines, due to high sensitivity to changes in culture conditions and generally require addition of cytokines IL-2 or IL-15 to support proliferation. NK cell lines also require irradiation prior to transfer to patients due to their malignant nature, which can be detrimental to the survival and cytotoxic effects of the transfused NK cells [82]. Nevertheless, NK-92 cells have gained FDA approval for clinical use and by 2021 were the most frequently used source of engineered NK therapies in clinical trials (43% of trials), followed by PB NKs (21%), iPSCs (17%), and CB NKs (13%) [48, 83].

NK-92 cells were derived from a 50-year-old male patient in 1994 with non-Hodgkin’s lymphoma and are considered an NK-like cell line due to expression of CD56 and many major NK activating receptors including NKp30, NKp46, 2B4, NKG2D and CD28. Conversely, NK-92 cells lack almost all the KIR receptors and express few inhibitory receptors (NKGA/B, low levels of KIR2DL4, ILT-2) and do not express the CD16 receptor responsible for ADCC. The strong cytotoxic activity of NK-92 cells can be attributed to this receptor expression profile which triggers perforin-granzyme cytotoxic pathways when activated in addition to death receptor cytotoxic pathways lead by FasL, TRAIL, tumor necrosis factor-like weak inducer of apoptosis (TWEAK) and TNF-α [84, 85].

Several derivative cell lines have been developed to overcome limitations with native NK-92 cells and move towards the development of “off-the-shelf” CAR-NK cells. NK-92MI were engineered to produce IL-2, negating the need for supplementary IL-2 [86]. A first-in-man clinical trial (NCT02944162) of NK-92MI expressing a CD33 CAR in patients with relapsed and refractory acute myeloid leukaemia (AML) demonstrated the clinical safety profile of these cells. NK-92MI cells were lentivirally transduced with a CAR CD33.CD28.41BB.CD3z construct, expanded and co-irradiated (10 Gy) prior to infusion. Following infusion of up to 5 × 10^9^ cells per patient, no significant adverse effects were observed although one patient experienced grade I CRS. One of the three patients treated did not respond to the treatment, while two of the three responded, but relapsed after 1 month and 4 months [87]. Although this was a small study with a limited number of patients, it was useful for demonstrating the positive safety profile of CAR-MI cells, but treatment outcomes were underwhelming.

Further optimisation of the NK-92 cell line resulted in production of high affinity NK-92 (haNKs) which express the CD16 receptor, increasing the ADCC activity [88]. Targeted affinity NK-92 (taNK) have been modified to target specific tumour cell ligands and furthermore t-haNKs combine all these features to express high affinity CD16, secrete IL-2 and recognise specific tumour ligands such as PD-L1 [89–91]. The potential of t-haNKs for treatment of characteristically difficult-to-treat pancreatic cancer was highlighted following the phase II QUILT 88 trial (NCT04390399) investigating ImmunityBio’s Nant Cancer Vaccine, comprising a combination of PD-L1 targeting t-haNK cells with an IL-15 receptor agonist Anktiva (N-803), aldoxorubicin, an albumin-modulated agent, plus low-dose chemotherapy in treatment of pancreatic cancer [92]. Following combination therapy overall survival for third, fourth- and fifth-line patients was 5.8 months, which was nearly double that of the historical survival of 3 months, and serious adverse effects were uncommon at 8%. With many more trials ongoing on CAR-expressing NK-92 cells investigating treatment of both haematological and solid tumours using a range of cancer targets including CD19, MUC-1, HER2, ROBO1 and PD-L1 [48], these early positive results may be an indication of the future potential of CAR-NK therapies exploiting NK-92 cells.

The KHYG-1 cell line has been shown to be more potently cytotoxic than NK-92 cells [93]. This suggests this cell line could also have therapeutic application as an “off-the-shelf” CAR-NK therapy as reported in a proof-of-concept study by Stikvoort et al. [94]. KHYG-1 cells were retrovirally transduced with a CD38-CAR containing a CD28 costimulatory domain and produced effective CD38-dependent cytotoxicity towards CD38^high^ multiple myeloma (MM) cell lines (UM9 and THP-1) and primary MM cells derived from patients. Furthermore, CD38-CAR KHYG-1 cells significantly slowed the growth of UM9 tumours in a xenograft model with a humanized bone marrow-like niche. RAG2^–/–γc–/–^ mice implanted with luciferase expressing UM9 MM tumours were treated with multiple i.v. injections of CD38-CAR KHYG-1 cells and at 20 days post treatment, only a fourfold increase in bioluminescence was detected in CD38-CAR KHYG-1 treated mice compared to 24-fold increase in the untreated cohort. This study highlights the potential for KHYG-1 cells to be developed into CAR-NK cells in a similar fashion to NK-92 cells, but further investigation is required to assess this in humans as results in mouse models do not always translate to humans.

NK cells lines which are derived from malignant sources require irradiation before clinical application, which can impact on proliferation and persistence in vivo. For example, NK-92 cells have a short lifespan in vivo, typically two days after initial infusion [95], which can be advantageous in terms of reducing side effects when compared to CAR-T therapies which can circulate for months or years after infusion. However, this short lifespan may result in inferior therapeutic outcomes and the requirement for multiple treatments. Already, NK-92 cells have been engineered to express IL-2 which enhances in vivo expansion, but more sophisticated engineering is required to combat NK exhaustion in the TME, particularly for treatment of solid tumours. KHYG-1 cells strongly express the inhibitory receptor NKG2A and when co-cultured with MM cells were found to express high levels of PD-1 [94]. This could be a major stumbling block in the development of CAR-NK therapies and will need to be addressed before such therapies are successful.

### CAR construct design for NK cells

**Fig. 4:**
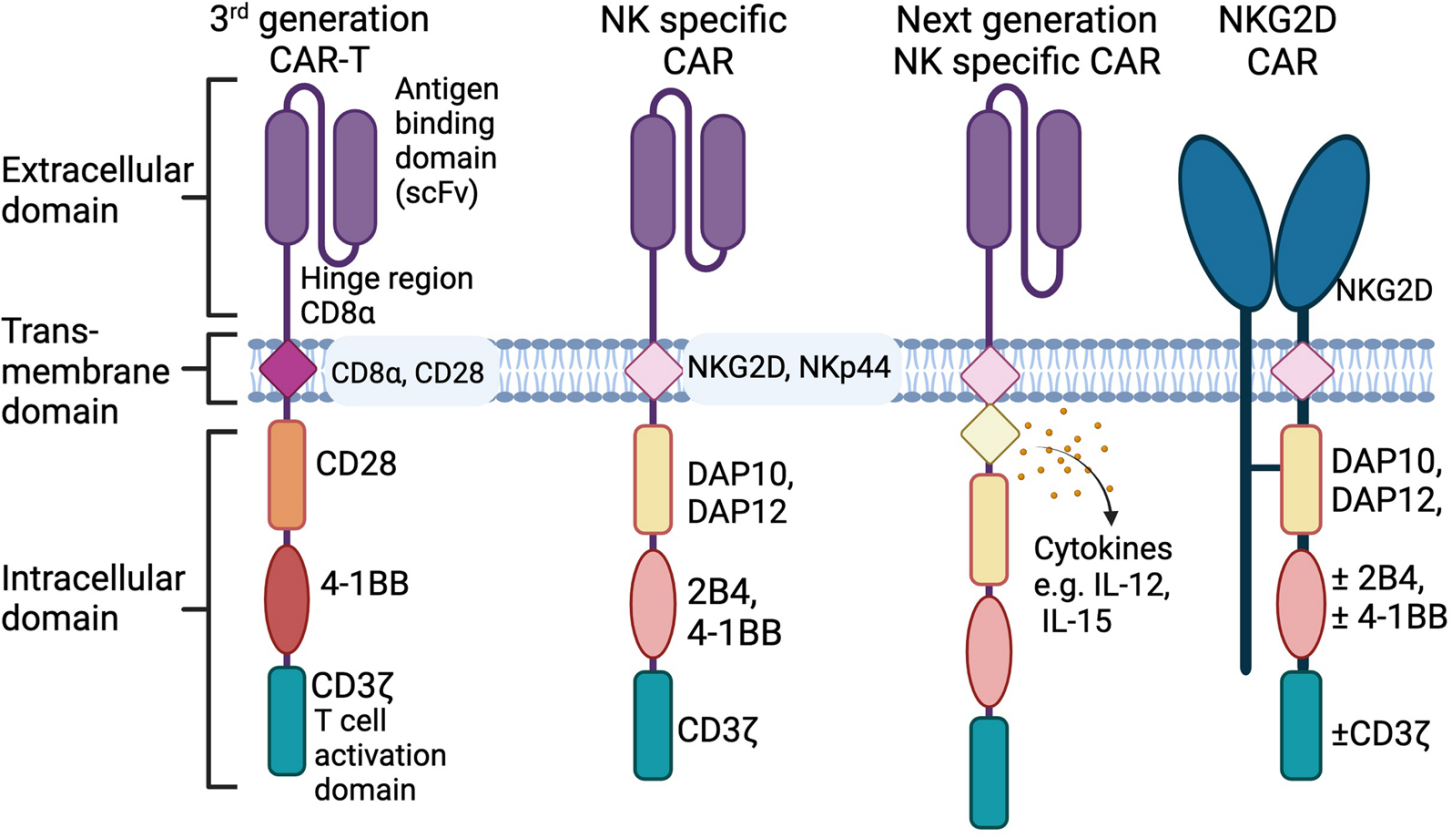
Schematic comparing CAR construct design elements for CAR-T and CAR-NK therapy. Initial studies investigating CAR-NK cells used the same constructs used for third generation CAR-T therapies, which are designed to mimic the T cell receptor. NK specific CAR constructs have since been developed to incorporate NK transmembrane signalling domains along with co-stimulatory receptors (e.g., DAP10, 2B4, CD28 or 4-1BB), to enhance NK cell effector functions. The activating receptor NKG2D has also been incorporated into NK-specific CAR constructs; although not a classical “CAR” as NKG2D is not an scFv, but they are referred to as CARs in the literature. Created with BioRender.com

Initial CAR-NK studies adopted the CAR-T backbone which had been designed to mimic the T-cell receptor. However, NK cytotoxicity is regulated by a distinct set of activating and inhibitory receptors which can be incorporated into CAR design to engage native NK receptors and exploit NK functionality. CAR-NK cells may be engineered to optimise NK cell effector functions by expressing an antigen-specific CAR molecule which incorporates NK-specific transmembrane signalling domains along with co-stimulatory receptors (e.g., NKG2D, DNAM-1, 2B4, CD28 or 4-1BB). These are designed to enhance NK cell activation upon recognition of tumour antigens and improve persistence, cytotoxicity, and cytokine production capabilities of CAR-NK cells (Fig. 4) [96–98].

Li et al. screened several CAR constructs optimised for activity in NK cells comprising various combinations of the transmembrane and co-stimulatory domains [73]. Activity of iPSC-NK cells expressing a CAR targeting mesothelin comprising a NKG2D transmembrane domain, 2B4 co-stimulatory domain, and CD3ζ signalling domain (termed CAR-iPSC-NK) were compared to a T-cell based CAR comprising two CD28 domains with 4-1BB (CD137) and CD3ζ (termed T-CAR-NK) in a luciferase-expressing A1847 ovarian cancer xenograft model. CAR-iPSC-NK resulted in significantly reduced tumour burden following bioluminescent imaging and improved survival when compared to the T-CAR-NK treatment group. Furthermore, following a comparison of CAR expressing primary T cells, survival was improved with 80% survival at day 70 in the CAR-iPSC-NK group compared to 20% survival in T-CAR group. Importantly, the T-CAR group exhibited evidence of severe side effects including significant weight loss (> 50%, n = 2 on days 23 and 39), severe visceral haemorrhage and ischemia, enlarged spleen and evidence of pathogenic damage to internal organs including liver, lungs, kidneys, and gut. In comparison, no such effects were observed in the CAR-iPSC-NK treatment group.

We know that NKG2D is a major activating receptor responsible for provoking caspase-mediated apoptosis following recognition of “kill me” stress signals expressed on tumour cells namely MICA, MICB and UL16 binding protein (ULBP-1 and ULBP-6) [26]. Exploiting NKG2D could enhance NK antitumour cytotoxicity. Xiao et al. report a small clinical trial investigating PBMC-derived NK cells expressing NKG2D fused to DAP12 co-stimulatory domain which was delivered by intraperitoneal (i.p.) infusion to three patients with metastatic colorectal cancer (NCT03415100) [99]. The authors report reduction of ascites generation and marked decrease in tumour cells in ascites samples in two of the three patients, and in the third patient rapid tumour regression in the liver region was observed with ultrasound and complete metabolic response confirmed by PET-CT scanning. All three patients were reported to have stable disease and importantly, no dose-limiting toxicities or GVHD were observed. Although the CAR constructs used in this study had a first-generation design with only one intracellular domain, it paves the way for further development of next generation CAR constructs bearing NKG2D receptors. This highlights the importance of careful NK-specific CAR construct design to maximise CAR-NK therapeutic effect and reduce side effects when compared to CAR-T constructs, and there is still a considerable number of NK receptors, co-stimulatory domains, and combinations of each to be explored.

### Genetic modification methods for CAR-NK manufacture

For large scale manufacture of an “off-the-shelf” CAR-NK product, the method used to genetically engineer the NK cells is an important consideration which can impact on production time, costs, and scalability. Ideally, the method of transfection for the creation of CARs should be efficient, scalable, and non-immunogenetic [100–102]. Currently, the majority of CAR therapies are engineered using viral vectors, which produce stable gene expression and higher transduction rates than non-viral methods [78]. NK cells are exceptionally challenging to transduce, particularly primary PB and CB NK cells, even with viral vectors which are usually very efficient at transducing a wide range of cells; owing to high expression of immune receptors, such as pattern recognition receptors (PRRs), which trigger apoptosis of NK cells following viral transduction [68, 103, 104]. Consequently, viral vectors commonly used in genetic editing for CAR-T production such as the vesicular-stomatitis-virus-G protein (VSV-G) lentivirus have reduced transduction rates (< 10% transduction efficiency) in NK cells. In the case of VSV-G, this is reportedly due to low expression of the low-density lipoprotein receptor on NK cells, required for VSV-G cellular entry [96].

The baboon envelope pseudotyped lentiviral vector (BaEV-LV), which binds to sodium-dependent neutral amino acid transporter (ASCT1 and ASCT2) for cellular entry, has been reported to produce enhanced transduction of 23% in freshly isolated human NK cells with a sustained transgene expression for at least 21 days [105]. For ex vivo genetic engineering, stable expression with lentiviral vectors is an important consideration, as cells are engineered to permanently express the transgene and then expanded before infusion into a patient [102]. However, viral vectors have significant disadvantages including safety concerns regarding mutagenesis, toxicity, immunogenicity; expensive time-consuming production which is in addition to the lengthy NK cell expansion protocols; and limited capacity for nucleic acids carriage which can be problematic when delivering larger 3rd, 4th and 5th generation CAR constructs [102, 106, 107]. For example, a 2nd generation CAR construct for lentiviral production could be around 10 kbp, with this increasing towards 20 kbp constructs as more components are added in for later generations [108]. Consequently, there is a need to develop safer, more efficient non-viral genetic modification methods for CAR-NK production.

## Gene editing tools for CAR-NK cells

Developments in genetic editing tools such as DNA transposons and Clustered Regularly Interspaced Short Palindromic Repeats (CRISPR) technologies have provided an alternative non-viral method of stable transgene expression. Transposon systems are a class of genetic elements with the ability to move or ‘transpose’ from one location to another within the genome via a ‘cut-and-paste’ mechanism, making them a powerful tool for genetic engineering [109]. The most promising transposons for gene therapy are derived from *Sleeping Beauty* (*SB*) and *piggyBac* (*PB*) systems [110]. Transposon systems comprise a two-component system with the transposon containing the gene of interest usually in plasmid form and the corresponding transposase enzyme which may be in the form of the protein or mRNA encoding the transposase protein. The transposon is flanked by specific DNA sequences known as inverted terminal repeats (ITRs) which are recognised by transposase. Upon recognition of the ITR, the transposase cuts out the transposon which is then inserted at a new genomic location [109]. Despite the potential of transposons systems for stable gene delivery, no transposon system has made it to the clinic yet due to concerns over unintended genomic disruptions or insertional mutagenesis and immunogenicity leading to difficulties gaining regulatory approval. Recent refinements in production of mini-circle vectors, developed for *SB* systems, which have minimal expression cassettes and lack the bacterial plasmid backbone, have helped to overcome issues including variable transfection efficiency and toxicity to host cells, associated with plasmid DNA vectors,and when paired with mRNA transposase has helped advance transposons towards the clinic [111]. The TcBuster transposon system from BioTechne is an example which has been developed specifically for CAR-T and CAR-NK production. It comprises a nanoplasmid containing a gene expression cassette paired with mRNA encoding the transposase enzyme. TcBuster delivered a CD19-CAR construct which also expressed EGFP to primary human PB NK and T cells successfully, resulting in CAR expressing NK and T cells which produced significantly more cytokines (IFNγ, TNFα and CD107a degranulation marker) than CAR-negative controls and efficiently killed target CD19 expressing Raji cells [70]. As of November 2023, seven clinical trials investigating CAR-T cells utilising transposon systems are registered on clinicaltrials.gov, indicating the potential for this technology to be applied in CAR-NK production [109].

CRISPR are classes of repeated DNA sequences that act in coordination with CRISPR-associated (Cas) genes. CRISPR technology comprises three main components: crispr-RNA which is complementary to the target gene; the Cas nuclease protein which is an enzyme guided by the crispr-RNA to the specific location on the DNA where it acts like molecular scissors to cut the DNA; and tracer-RNA which helps in processing and binding of the crispr-RNA and Cas nuclease [112]. CRISPR technology has been employed to enhance the cytotoxicity of the NK-92 cell line which has less potent immune functions when compared to primary NK cells [113]. CRISPR engineering with Cas9 ribonucleoproteins (RNP) complexes targeting *CD96* and *KLRC1* (encoding NKG2A) was delivered by nucleofection to successfully knockout inhibitory CD96 and NKG2A receptors resulting in 2.8% of edited NK-92 cells expressing these receptors compared to 92.5% unedited parental cells. In addition, the authors knocked-in a fluorescent mCherry gene and replaced a silenced promoter to reactivate endogenous CD16 and DNAM-1. The resultant CRISPR-engineered NK-92 cells demonstrated markedly enhanced cytotoxicity compared to unedited NK-92 cells and could mediate ADCC (via CD16) against a range of cancer cell lines including MDA-MB-231 and BT-474 breast cancer cells. This study highlights the potential for CRISPR in engineering to exploit the various NK activating and inhibitory receptors for therapeutic benefit.

Transposons and CRISPR technology may also be used concomitantly to combine powerful genetic editing activity. Indeed, a combination of transposon engineering and CRISPR/Cas9 genome editing was used to generate potent CAR-NK cells expressing the myeloid associated antigen CLL-1 in PB NK cells [71]. The TcBuster transposon system delivered the CLL-1 CAR construct, while CRISPR/Cas9 cargo was applied to knockout the NK cell cytokine checkpoint cytokine inducible SH2-containing protein (CIS, product of the *CISH gene*). Concurrent *CISH* knockout in CLL-1 CAR-NK cells were associated with reduced DNAM-1 and increased CD69 expression, reduced expression of NKG2D, NKp30, NKG2A, PD-1 and TIGIT. Enhanced primary AML blast cytotoxicity was observed of both control and CLL-1 CAR-NK cells indicating the potential for these technologies to enhance cytotoxicity and alter NK cell phenotype. Similarly, this *CISH* knockout approach has been employed in human iPSC-derived NK cells [114], and in CB-derived NK cells [115]; resulting in enhanced in vivo persistence, metabolic fitness and antitumour activity. These studies serve as examples of the potential for non-viral genetic editing but the complexity of the balance between NK activating and inhibitory receptors requires much further study to optimise and exploit this therapeutically.

It is also important to acknowledge the safety and ethical concerns that exist regarding the risk of insertional mutagenesis with gene editing tools including both transposon and CRISPR technology. Transposon genomic integration runs the risk of potential activation of oncogenes or interruptions of tumour-suppressor gene which may contribute to malignant transformation. Although *SB* transposon systems have demonstrated a good safety profile so far, a clinical trial employing *PB* engineered donor derived CD19 CAR-T cells resulted in 2 out of 10 patients developing T cell lymphoma derived from the CD19-CAR-T cells [116]. Despite the potential of this technology, further research is required to ensure the safety of transposon systems before progress to the clinic may be achieved. Strategies including the avoidance of strong viral enhancers which may increase the risk of oncogene activation, employment of “safe harbor” sites and site-directed DNA binding domains to enable site-specific transpositions are potential solutions to overcome problems with transposons [109, 117]. In the case of CRISPR, “off-target” DNA cuts may introduce unwanted mutations and potentially exacerbate retrotransposition [118]. Approaches to reduce off-target effects include optimisation of different components of CRISPR-Cas9 systems, development of prime editors and RNA editing [119]. Nevertheless, in November 2023 the UK Medicines and Healthcare Products Regulatory Agency (MHRA) was the first to approve a CRISPR-Cas9 gene editing therapy, Casgevy (exagomglogene autotemcel), for treatment of transfusion-dependent β-thalassemia and sickle cell disease. This landmark approval was followed by the FDA in December of Casgevy and Lyfgenia (lovotibeglogene autotemcel) and European Medicines Agency (EMA) approval of Casgevy [120]. Although patients treated with Casgevy and Lyfgenia are still undergoing long-term evaluation on safety and effectiveness of these treatments, this approval will undoubtedly open the door for further use and approval of CRISPR genome editing technologies.

## Non-viral gene delivery to NK cells

**Fig. 5:**
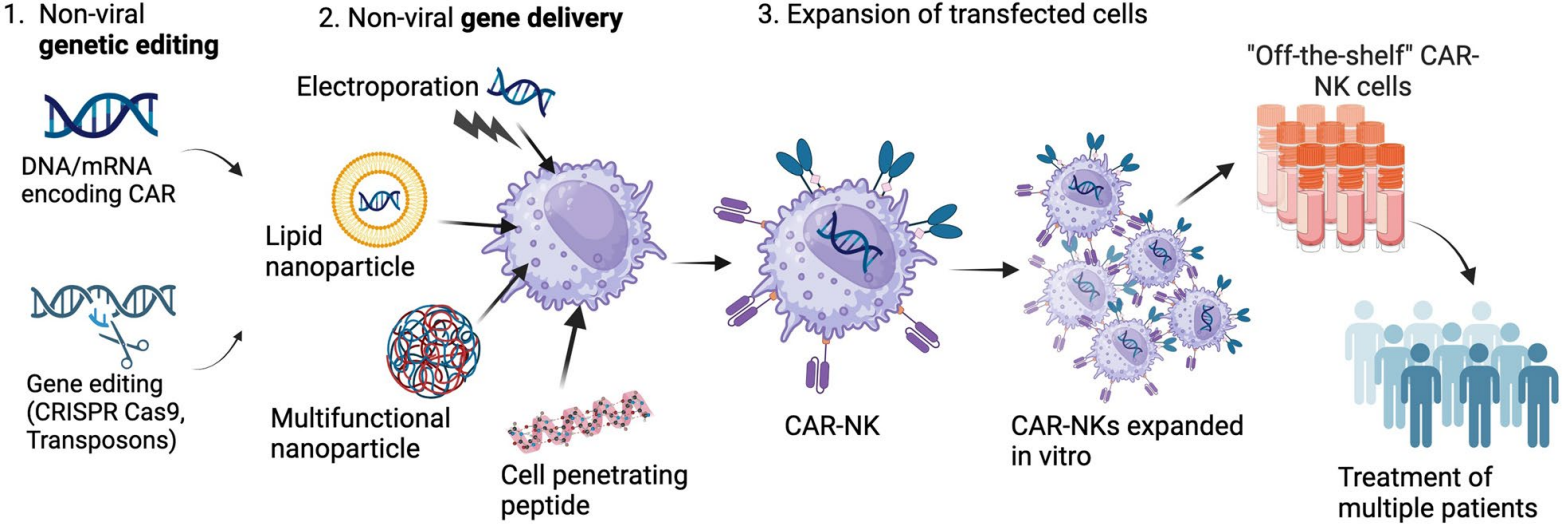
Non-viral genetic engineering of “off-the-shelf” CAR-NK cells requires efficient transfection and stable expression of the CAR transgene. 1. Introduction of CAR constructs encoded in DNA and/or mRNA or genetic editing using CRISPR Cas9 or DNA transposon technologies such as *PiggyBac* and *Sleeping Beauty* to give stable gene expression. 2. Nucleic acids encoding the CAR construct may be delivered to NK cells via several non-viral delivery strategies including electroporation, lipid nanoparticles, cell penetrating peptides or multifunctional nanoparticles. 3. Following transfection, NK cells expressing the CAR construct are expanded in vitro to produce an “off-the-shelf” CAR-NK product which may be used to treat multiple patients

Despite the huge potential for non-viral genome editing methods, a major hurdle is the method of intracellular delivery. Ultimately, transposon and CRISPR technologies are comprised of nucleic acids whether this is in DNA or RNA form, and thus require a method of cellular entry. In general, the most common non-viral delivery methods employed are via physical electroporation of naked nucleic acids, or chemical methods involving complexation with lipids, cationic polymers, proteins, or cell penetrating peptides (CPPs) which package nucleic acids and facilitate intracellular delivery, summarised in Fig. 5. Despite success in other cell types, NK cells have proven more difficult to transfect, with disappointing non-viral transfection rates compared to viral transduction. For ex vivo gene delivery, the main barriers to gene delivery are the cell membrane, endosomal entrapment and in the case of DNA, entry to the nucleus [106], for successful gene delivery an ideal delivery vector must overcome each of these barriers while being non-toxic.

Electroporation (including nucleofection) is the most widely employed method for non-viral delivery to NK cells with transfection efficiency rates up to 90% [121], but toxicity can be a problem due to the strong electrical field applied to cells and can increase manufacturing timescale and costs. Cell viability has been reported as low as 2% following electroporation of primary NK cells for delivery of a plasmid encoding the reporter gene green fluorescent protein (GFP), although with careful optimisation this was increased to 50%, and some electroporator manufacturers quote viability rates of 80–90% [122, 123]. Nevertheless, these methods require careful optimisation, cytokine stimulation or expansion with feeder cells for adequate transfection efficiency and viability [99, 124]. A small number of studies have utilised non-viral delivery systems as the method of gene delivery for NK cells (summarised in Table 3), resulting in varying degrees of success.

**Table 3:** Summary of non-viral delivery systems for gene delivery to NK cells

Name	Type of delivery system	Details of delivery system components	Cargo	NK cell source	Transfection efficiency	Cell viability	Source
YSK12-C4	Lipid based “MEND”	Ionisable-cationic lipid containing unsaturated carbon chains: YSK12-C4 lipid (6Z, 9Z, 28Z, 31Z)-19-(4-(dimethylamino)butyl) heptatriaconta-6,9,28,31-tetraen-19-ol) PEG-DMG: 1,2-dimyristoyl-sn-glycerol methoxyethyleneglycol 2000 ether. Prepared in 85/15/1 molar ratio of YSK12-C4, cholesterol and PEG-DMG respectively.	siRNA GAPDH	NK-92 cell line	75%	60%	[125]
Lipid5/DSPC/ β-sitosterol/ DMG-PEG2K)	LNP	Ionisable “Lipid 5”, 1,2-distearoyl-sn-glycero-3-phosphocholine (DSPC), β-sitosterol and 1,2-Dimyristoyl-rac-glycero-methoxypolethelene glycol-2000 (DMG-PEG2K)	mRNA GFP (5moU modified)	KHYG-1 cell line	>80%	>95%	[126]
				CB NK cells	>75%	>95%	
CL1H6	LNP	CL1H6 has the same hydrophilic head group as YSK12-MEND and an oleate structure as a hydrophobic tail, with an amino moiety in place of a methyl group Prepared in 25/75 ratio CL1H6 and cholesterol.	siRNA GAPDH	NK-92 & KHYG-1 cell lines	90%	90%	[127]
CART	LNP	Multiblock oligomers (poly(carbonate)-b-O(α amino ester)s) consisting of ≥1 lipid block and a charge-altering block via ester-to-amide rearrangement of the cationic poly(α amino ester) backbone into neutral small molecules (diketopiperizine).	mRNA GFP (5meC modified)	PB NK (primary)	32%	78%	[128]
MF-NPs	Magnetic core NPs	Zn/Fe core shell capped with caffeic acid and coated with cationic polydopamine polymer	Plasmid CAR (anti-EGFR)	NK-92MI cell line	60%	98%	[129]
PAGE	Peptide-mediated	Cas9 fused at N-terminal to HIV TAT peptide (GRKKRRQRRR) and 4x Myc nuclear localisation signals (NLS). 2x SV40 NLS and GFP at C-terminal Co-delivered with ‘assist peptide’; a TAT-HA2 fusion peptide (RRRQRRKKRGGDIMGEWGNEIFGAIAGFLG)	CRISPR Cas9	NK-92 cell line	78%	>80%	[130]

### Lipid-based delivery to NK cells

Lipids have been employed in liposomes and lipid nanoparticles (LNP) to deliver nucleic acids for a range of applications, including most significantly, the use of lipid nanoparticles to deliver the mRNA covid vaccine by both BioNTech and Moderna [131]. The approval of these LNP-based vaccines represents a significant breakthrough for the field of non-viral delivery systems and has paved the way for more to follow. Lipids are amphiphilic molecules containing a polar head group linked to a hydrophobic tail and lipids with a range of properties have been employed for gene delivery. Cationic lipids such as 1,2-di-*O*-octadecenyl-3-trimethylammonium-propane (DOTMA) and its derivative 1,2-dioleoyl-3-trimethylammoium-propane (DOTAP) allow packaging of genetic cargo and intracellular delivery through electrostatic interaction and membrane fusion. Ionisable lipids are neutral at physiological pH which become protonated at low pH in endosomes, facilitating endosomal release of cargo. Other lipids such as 1,2-distearoyl-*sn*-glycero-phosphocholine (DSPC) and cholesterol analogues have been employed to help stabilise LNP structure [132]. LNPs may be internalised via various endocytic pathways such as macropinocytosis, clathrin-mediated and caveolae-meditated endocytosis and this differs between cell types. Following internalisation, LNPs usually enter the endosomal compartment and require an escape mechanism for cargo release [133]. As a result, combinations of lipids with different properties are often employed to optimise LNP functionality.

Nakamura et al. developed a multifunctional envelope-type nanodevice (MEND) containing an ionisable-cationic lipid to facilitate endosomal escape, termed YSK12-C4 lipid (YSK12-MEND), for delivery of siRNA to immune cell lines [125]. The authors demonstrated the transfection capacity of the YSK12-MEND in a range of human immune cells delivering siRNA encoding GAPDH. Gene silencing in NK-92 cells was 75% which was much higher than commercially available Lipofectamine RNAiMAX at 19% silencing. However, the YSK12-MEND LNP caused cytotoxicity in NK-92 cells (60% viability at 30 nM siRNA dose). The same group recently produced LNPs comprising a pH-sensitive cationic lipid termed CL1H6 for siRNA delivery to NK cell lines [127]. CL1H6 has the same hydrophilic head group as YSK12-MEND and an oleate structure as a hydrophobic tail, with an amino moiety in place of a methyl group, which facilitated cellular uptake and endosomal escape in NK-92 cells via membrane fusion. The ratio of CL1H6 and cholesterol in the LNPs was optimised at 25/75 to give better gene silencing effects (90% gene silencing of GAPDH following delivery of siRNA targeting GAPDH), while maintaining good cell viability (90% viability) in NK-92 cells. Similar results were also reported for delivery to KHYG-1 cells. The authors attribute this improved cell viability to the membrane fusion activity during endosomal escape. YSK12-MEND escapes endosomes with a high degree of membrane fusion, while CL1H6-LNP escaped endosomes with only mild membrane fusion. Similarly, Douka et al. recently optimized LNP formulations for mRNA delivery to NK cells with ionisable lipids [126]. The optimized LNPs comprised ionisable “Lipid 5”, DSPC, β-sitosterol and 1,2-Dimyristoyl-rac-glycero-3-methoxypolyethelene glycol-2000 (DMG-PEG2K) produced > 80% transfection efficiency in the KHYG-1 cell line and 75% in CB derived NK cells, with > 95% cell viability in each cell type.

Wilk et al. present the use of charge-altering releasable transporters (CARTs) for gene delivery to NK cells [128]. CARTs are multiblock oligomers consisting of ≥ 1 lipid block and a charge-altering block designed for mRNA delivery [134]. CARTs are initially cationic to complex anionic nucleic acids but biodegrade to neutral diketopiperazine small molecules under physiological conditions (pH7.4), facilitating release of the anionic cargo. Following transfection with CART delivering 31 ng mRNA encoding GFP to PB NK cells, 32% of cells expressed GFP with 78% viability, which was significantly better than Lipofectamine 2000 (0% GFP^+^ cells, 90% viability) and electroporation (2% GFP^+^ cells, 40% viability) (N.B. when the mRNA mass delivered was increased to 10 μg via electroporation transfection efficiency rose to 28% GFP^+^ cells, with 40% viability). Furthermore, CART successfully generated CD19-CAR-NK cells which were potently cytotoxic against CD19^+^ Nalm6 cells with 10% dead target cells compared to 5% with naïve NK cells. This demonstrates the potential of CARTs as a non-viral system for CAR-NK generation, but transient mRNA delivery could pose a problem for expansion and efficacy of CAR-NK cells in vivo. Further studies are required to expand this method for larger in vivo studies, assess the lifespan of CART-generated CAR-NK cells in vivo and compare efficacy to virally transduced CAR-NK cells.

### Multifunctional nanoparticles for delivery to NK cells

Kim et al. described multifunctional nanoparticles (MF-NPs) comprising a core magnetic Zn/Fe shell, capped with caffeic acid and coated with cationic polydopamine polymer on the surface which form complexes with anionic nucleic acids for CAR delivery to NK-92MI cells [129]. The magnetic core enabled magnetic resonance imaging of NK cells and facilitates in vivo monitoring of cell trafficking. A plasmid encoding an epidermal growth factor receptor (EGFR) CAR construct was delivered by the MF-NPs to NK-92MI cells with a transfection efficiency of 60% and cell viability of 98%. Following tail vein injection of EGFR-CAR-NK-92MI cells to NK cell-free NOG mice (NOD/Shi-*scid*/IL2Rγ^null^) mice bearing MDA-MB-231 xenografts, tumour growth was significantly less in the CAR-NK-92MI group compared to mice treated with naïve NK-92MI and PBS control (twofold and 3.5-fold difference in tumour size respectively). Further work would be required to assess the biocompatibility and degradation profile of magnetic based NPs and ensure adequate expansion and persistence of MF-NP edited CAR-NK cells for clinical application.

### Peptide-based delivery to NK cells

Peptide-based vectors have gained attention for gene delivery due to advantages including biocompatibility, flexibility and low toxicity [135]. Peptides with a wide range of specific functions have been derived from viruses or designed to mimic viral vector sequences which enable and enhance gene delivery and are generally classified according to function; DNA condensing peptides, CPPs, endosmolytic peptides and nuclear location sequences (NLS). CPPs are short peptides (5–30 amino acids) which efficiently carry macromolecular cargo across the cell membrane, without the need for receptors or other carriers [136]. A peptide-assisted genome editing (PAGE) CRISPR-Cas system has been developed which utilises CPP and endosomal escape peptides for genome editing in myeloid cells, primary T cells and NK-92 cells [137]. A cell penetrating Cas9 was designed which comprised Cas9 fused N-terminally to HIV TAT (GRKKRRQRRR) and 4 × Myc NLS, and C-terminally with 2 × SV40 NLS and GFP. This cell penetrating Cas9 was then co-delivered with an ‘assist peptide’; a fusion peptide (termed TAT-HA2 (RRRQRRKKRGGDIMGEWGNEIFGAIAGFLG)) comprising the CPP TAT peptide derived from HIV-1 and an endosomal escape peptide from influenza A virus hemagglutinin (HA2) protein. The TAT-HA2 assist peptide potentiated Cas9 editing in mCherry^+^ EL4 T lymphoblast reporter cells from undetectable levels to 85% efficiency with over 80% cell viability. Furthermore, the PAGE system resulted in 78% gene editing in NK-92 cell line, highlighting the potential of CPPs for gene editing in NK cells. However, this is a complex engineering strategy and would require much more development if a CAR construct was to be delivered.

## NK cell specific challenges to gene delivery

The current difficulties in non-viral transfection of NK cells are compounded by a lack of understanding of the specific biological barriers in these highly specialised cells and elements which are specific to NK cells much be considered in the design of non-viral delivery systems for NK cell therapies. Cellular uptake is the first biological barrier for gene delivery and NK cells with specialised activating and inhibitory receptors are more complicated than most cell types. Following intracellular uptake, endosomal escape is the first hurdle for successful gene delivery. Considering the specialised lytic machinery of NK cells is important to avoid degradation of genetic cargo in the endosomal/lysosomal compartment. Furthermore, the innate immune activity of NK cells via PRR signaling which facilitates NK anti-viral activity has been cited as a reason for NK cell resistance to viral vectors, and non-viral vectors must be designed to avoid this same fate. Overcoming these NK-specific barriers to gene delivery will be integral for a non-viral gene delivery system is to be successful in NK cell therapies.

### Intracellular delivery to NK cells

Endocytosis is thought to be the main uptake pathway for most non-viral gene delivery systems and may be categorised as clathrin-mediated, caveolae-mediated, or micropinocytosis. Uptake may be influenced by factors including properties of the delivery system and the cell type [106, 132]. In the case of NK cells, the balance of activating and inhibitory receptors is regulated by endocytosis and recycling of receptors to the cell surface. For example, inhibitory receptor CD94/NKG2 continuously recycles between the cell surface and intracellular compartments in a process which resembles macropinocytosis [137]. Influenza virus directly infects PBMC-NK cells via clathrin and caveolin-dependent endocytosis [138]. Infection occurs by binding of viral HA protein to α-2,3 and/or α-2,6 linked terminal sialic acids, which are present on the surface of NK cells and specifically on activating receptor NKp46. The interaction of NKp46 with HA on the surface of virus-infected cells is a known mechanism for NK cell recognition and killing of infected target cells, but it appears that the virus itself can also infect NK cells through interaction with NKp46 [139], which results in increased apoptosis of infected NK cells, and decreased NK cytotoxic activity. This knowledge of viral infection methods may be exploited for the design of non-viral delivery systems, in terms of targeting or avoiding specific uptake pathways to enhance gene delivery, without unwanted activation causing phenotypic changes within the NK cells.

Cationic delivery systems, and particularly arginine-rich peptides are known to electrostatically bind to anionic species present on the extracellular surface of the cell membranes (e.g. lipid head groups, heparan sulphate proteoglycans (HSPGs) and syndecans on the cell membrane, initiating cellular uptake [140]. Letoha et al. report that syndecans facilitate uptake of cationic CPPs (e.g., TAT) [141]. The study revealed that syndecan-4 particularly binds and mediates transport of cationic CPPs through the plasma membrane into K562 erythroleukemia cells and higher syndecan-4 levels is correlated with higher transfection efficiency. The amount and size of heparan sulfate chains present on syndecans varies depending on the different stages of cellular differentiation and cell type [142]. Therefore, the expression of syndecans by different cells may lead to varying transfection results. HSPGs and syndecans are expressed by NK cells and have been reported to interact with activating NCRs (NKp30, NKp44 and NKp46) and KIR2DL4 (inhibitory receptor) and modulate their function [143–145]. These receptors can recognise and bind HSPG in *trans* which are often upregulated in target cells (e.g. cancer cells), but they may also bind with HSPG in *cis* (on the surface of the NK cells); impacting the function of the NK receptors through masking interactions with target cells or may affect the trafficking of NCRs to intracellular degradation and recycling pathways. If a delivery system binds to HSPG or syndecans for endocytosis, there is a risk that this will trigger unwanted activation of NCRs; impacting the activation status of the NK cells or trigger apoptosis. Therefore, careful design is required to give the best transfection efficiency without perturbing NK receptor status.

Receptor-mediated endocytosis is commonly employed to enhance transfection of gene delivery systems and active targeting strategies may help to overcome problems with HSPG-linked endocytosis. Again, inspiration may be taken from viral vectors when identifying suitable targets for receptor-mediated endocytosis. For example, BaEV lentiviral transduction is mediated by binding with ASCT1 and ASCT2 in freshly isolated human NK cells [105], which could potentially be targeted by non-viral delivery systems.

### Endosomal Escape in NK cells

Non-viral delivery systems have been designed to facilitate endosomal escape through the “proton sponge” effect or fusogenic activity in response to low pH in endosomes [146]. However, the exact pH of endosomal compartment can vary depending on cell type, with differences between cell lines and primary cells, and the specific function or cargo content within the endosome [147]. There is little information in the literature regarding NK endosomal pH in the context of designing gene delivery systems. We can compare other immune effector cells such as T cells and macrophages to gain insight into immune cell endosomal characteristics, but this is no substitute for assessing NK cells specifically. Endosomal acidification is reported to be slower and not as robust in human Jurkat T cells (slowly drops from pH 6 to pH 5.2 at 240 min after uptake) compared to the model HeLa human cell line (pH drops to 5.0 within 120 min of uptake) commonly used to evaluate cationic polymers for gene delivery, with primary T cells showing higher endosomal pH (> pH 6) [148]. It has also been reported that early endosomal and recycling endosomes have a neutral pH in macrophages, which promotes the activity of transient receptor potential muclipin 2 (TRPML2); a calcium-permeable cation channel resident in the lysosomal compartment involved in recycling of plasma membrane proteins [149]. TRPML2 is also expressed in CD57^+^ NKG2C^+^ NK cells and is speculated to be involved in modulating chemokine secretion in activated NK cells [150]. It could therefore be extrapolated that endosomes in NK cells are also likely to have a higher pH (pH > 6) at least in the early endosomal compartment, but studies are needed to confirm this. When considering NK cell cytotoxic function is exerted primarily through specialised secretory lysosomes, or lytic granules, containing perforin and granzymes [151], it is possible that the presence of this lysosomal machinery may therefore impact on endosomal trafficking and escape following transfection. If the pH of endosomes in NK cells is closer to neutral as with T cells and macrophages, then this could pose a problem for delivery systems requiring a lower pH to trigger endosomal escape and warrants further investigation to optimise the design of delivery systems specific to NK cells.

### Avoidance of pattern recognition receptor (PRR) activation in NK cells

During infection, NK cells detect the presence of viral or bacterial pathogen-associated molecular patterns (PAMPs) via PRRs, which are an essential component of the NK-cell mediated innate immune response. NK cells express a wide variety of PRRs including Toll-like receptors (TLR) 1–10, RIG1, NOD2 and MDA5 which are involved in the induction of cytotoxicity when activated [152]. TLR3, 7, 8, are expressed in endosomes and detect double stranded RNA (TLR3), single stranded RNA (TLR7 and 8) and CpG DNA (TLR9). This nucleic acid sensing facilitates virus detection and antiviral immune response [153]. Such PRRs have been cited as one reason for NK resistance to viral-based transduction. Indeed, inhibition of PRR activity resulted in increased transduction efficiency with a VSV-G pseudotyped lentiviral vector in NK cells. The authors pretreated freshly isolated PB NK cells with BX795, an inhibitor of the TBK1/1KKe complex which acts downstream of PRRs RIG-I, MDA5 and TLR3, which resulted in a 3.8-fold increase in lentiviral transduction efficiency. Similar pronounced enhancement of transduction in the presence of BX795 was also observed in the NK-92 cell line [154].

The nucleic acid sensing of PRRs therefore is a barrier to gene delivery to NK cells. Delivery of mRNA or DNA cargo is likely to activate PRRs triggering antiviral NK processes, resulting in poor viability of transfected NK cells. The use of modified mRNAs for genetic cargo may be utilized to avoid recognition of PRRs in NK cells. Substitution of nucleosides has proven to be significant in mitigating PRR detection and improves mRNA translation. Common modifications include uridine replacement with similar nucleosides such as pseudouridine (ψ), or N-1-methyl-pseudouridine (m1ψ), and cytosine can be replaced with 5-methylcytosine [155, 156]. The studies discussed in Sect. "Lipid-based delivery to NK cells" include two examples employing modified mRNA for delivery to NK cells. Douka et al. delivered 5-methoxyuridine (5moU)-modified mRNA with LNPs, while McKinlay et al. delivered 5-methylcytosine-modified mRNA. Neither study commented on their decision to employ modified mRNA or on the reasons for choosing the specific modification. Various substitutions have been employed, particularly within the mRNA vaccine field with pseudouridine-modified mRNA producing superior translation attributed to reduced binding with RNA-dependent protein kinase [157, 158]. Nevertheless, further research is required to define optimal nucleoside modification for CAR delivery to NK cells.

If DNA based cargo is to be delivered, for example in transposon systems, then limiting the CpG content should be considered. In the context of CAR construct delivery, this is particularly important considering the larger size of CAR constructs to be delivered. DNA minicircle technology has been employed in *SB* transposon systems to help reduce the size of cargo and enhance transfection efficiency. The TcBuster system uses a “nanoplasmid” technology which has been designed specifically for CAR construct delivery to T and NK cells [70].

## Conclusion and future directions

In conclusion, this review details a comprehensive analysis of the current state of CAR-NK therapies and non-viral engineering of NK cells, highlighting the potential of CAR-NKs as a promising therapeutic approach in the field of cancer immunotherapy, and challenges which need to be overcome in order to realise that potential. The use of CAR-NK therapies is still at an early stage, but potent anti-tumor activity has been demonstrated in preclinical and early-phase clinical trials across various cancer types. The enhanced safety profile and potential for a more scalable, cost-effective “off-the-shelf” CAR-NK therapy would increase accessibility to patients; improving patient outcomes and quality of life. Development of efficient non-viral engineering strategies specific to NK cells are required to improve manufacturing processes of CAR-NK therapies to support the growing demand for advanced therapeutics. To date, the ideal non-viral delivery system has yet to be developed which efficiently delivers CAR transgenes to NK cells without impacting viability or receptor status of the cell; given how important receptor status is for NK cell functionality. Further investigations into novel delivery systems and clinical trials are warranted to fully unlock the therapeutic potential of CAR-NK cells and translate these findings into routine clinical practice.

## Data Availability

No datasets were generated or analysed during the current study.

## Abbreviations

21spe-MLL5: Exon 21 specific mixed lineage leukemia protein-5
ADCC: Antibody-dependent cellular cytotoxicity
AICL: Activation inducted C-Type Lectin
ALL: Acute lymphoblastic leukemia
AML: Acute myeloid leukaemia
ASCT: Sodium-dependent neutral amino acid transporter
BaEV-LV: Baboon envelope pseudotyped lentiviral vector
BCMA: B-cell maturation antigen
CAR: Chimeric antigen receptor
CART: Charge-altering releasable transporter
Cas: CRISPR-associated
CB: Umbilical cord blood
CD3z: CD3 zeta chain
cDC1: Conventional type 1 dendritic cells
CIS: Cytokine inducible SH2-containing protein
CLL: Chronic lymphocytic leukaemia
CPP: Cell penetrating peptide
CRISPR: Clustered Regularly Interspaced Short Palindromic Repeats
CRS: Cytokine release syndrome
DMG-PEG2K: 1,2-Dimyristoyl-rac-glycero-3-methoxypolyethylene glycol-2000
DOTAP: 1,2-Dioleoyl-3-trimethylammonium-propane
DOTMA: 1,2-Di-O-octadecenyl-3-trimethylammonium propane
DSPC: 1,2-Distearoyl-sn-glycero-3-phosphocholine
EBV-LCL: Epstein-Barr virus-transformed lymphoblastoid feeder cells
EGFR: Epidermal growth factor receptor
EMA: European Medicines Agency
FDA: U.S. Food and Drug Administration
GAG: Glycosaminoglycans
GFP: Green fluorescent protein
GPI: Glycophosphatidylinositol
GREX: Gas-permeable static cell culture flasks
GVHD: Graft-versus-host disease
HA: Viral hemagglutinin
haNKs: High affinity NK92
HDR: Homology-directed repair
HER2: Human epidermal growth factor receptor 2
HLA: Human leukocyte antigen
HS: Heparan sulfate
HSPGs: Heparan sulphate proteoglycans
i.p.: Intraperitoneal
IFN-γ: Interferon-gamma
Ig: Immunoglobulin
IgG: Immunoglobulin G
IL-2: Interleukin-2
IL-2Rβ: Interleukin-2 receptor beta chain
iPSCs: Inducible pluripotent stem cells
ITAM: Immunoreceptor tyrosine-based activation motif
ITIM: Immunoreceptor tyrosine-based inhibitory motifs
ITR: Inverted terminal repeat
iwCLL: International Workshop on CLL
KIR: Killer Ig-like receptors
LBCL: Large B-cell lymphoma
LNP: Lipid nanoparticles
MCL: Mantle cell lymphoma
MEND: Multifunctional envelope-type nanodevice
MF-NPs: Multifunctional nanoparticles
MHC: Major histocompatibility complex
MHRA: UK Medicines and Healthcare Products Regulatory Agency
MICA/MICB: Major histocompatibility complex class 1 chain-related protein A/B
MM: Multiple myeloma
NCR: Natural cytotoxicity receptor
NHEJ: Non-homologous end joining
NK: Natural killer
NLS: Nuclear localisation signals
PAGE: Peptide-assisted genome editing
PB: Peripheral blood
PB: PiggyBac
PBMC: Peripheral blood mononuclear cells
PCNA: Proliferating cell nuclear antigen
PD-1: Programmed cell death protein
PDGF-DD: Platelet-derived growth factor-DD
PHA: Phytohemagglutinin
PRR: Pattern recognition receptor
PVR: Poliovirus receptor
r/r: Relapsed or refractory
RNP: Ribonucleoproteins
SB: Sleeping Beauty
scFv: Single-chain fragment variable
SLAM: Signalling lymphocyte activation molecule
t-haNKs: Targeted high affinity NK-92
TAA: Tumour associated antigen
taNKs: Targeted affinity NK-92
TCR: T-cell receptor
TIGIT: T-cell immunoglobulin and ITIM domain
TME: Tumour microenvironment
TNF-⍺: Tumour necrosis factor-alpha
TOL: Toll-like receptor
TRAIL: TNF-related apoptosis-inducing ligand
TRPML2: Transient receptor potential muclipin 2
TRUCK: T-cells redirected for universal cytokine-mediated killing
TWEAK: Tumor necrosis factor-like weak inducer of apoptosis
ULBP: UL16 binding protein
VSV-G: : Vesicular-stomatitis-virus-G protein
